# Book Reviews

**Published:** 1906-02

**Authors:** 


### BOOK REVIEWS.

Man and His Poisons. A practical exposition of the causes, symptoms and treatment of self-poisoning. By Albert Abrams, A. M., M. D., (Heidelburg) F. R. M. S., Consulting Physician to Denver National Hospital for Consumptives, Director of the Medical Clinic Cooper Medical College of San Francisco, etc. Cloth, 258 pages. Price $1.50. E. B. Treat & Co., Publishers, 241 W. 23rd St., N. Y., 1906.

The human body is a receptacle and laboratory of poisons, and man is at all times exposed to the danger of being overcome by poisons generated in his system. The subject of self-poisoning has advanced from a plausible and entertaining theory to an established fact, and Dr. Abrams in this excellent monograph goes into the subject very thoroughly and practically, giving in plain and correct terms the etiology, symptoms and treatment. Repeated reference is made to the principles of Psychotherapy, the mind being a most important factor for influencing the body for good or ill.

Blakiston's Quiz Compends. A Compend of Medical Chemistry, Inorganic and Organic, including Urinary Analysis. By Henry Leffmann, A. M., M. D., Professor of Chemistry in the Woman's Medical College of Pennsylvania, and the Wagner Free Institute of Science. Fifth edition, revised. 12mo, cloth, 200 pages. Price, $1.00. P. Blakiston's Son & Co., Publishers, 1012 Walnut St., Philadelphia, 1905.

A very excellent little compend that will be found of especial value to students of Medicine and Dentistry. We have had occasion to commend the previous editions and can say that this fifth edition is fully revised and brought up to the latest advances in Medical Chemistry. For correctness of statement and the clearness and conciseness of the text it may be relied on.

A Laboratory Manual of Physiological Chemistry. By E. W. Rockwood, B. S., M. D., Professor of Chemistry and Toxicology in the University of Iowa. Second Edition, revised and enlarged. Large 12mo, 229 pages, extra cloth. Price $1.00, net. F. A. Davis Co., 1914 Cherry St., Philadelphia, Publishers.

This book has been prepared with the aim of imparting accurate knowledge through the student's own observation. There has been included with the directions for experimental work a brief explanation of the facts observed, so as to call attention to their meaning.

The value of the work lies in the fact that the work covers the entire ground and is not limited to a few tests of the urine, as is usually the case. The student gains his knowledge from his own observations, from seeing and handling the substances which he studies. Brief explanations are given in order that he may draw the proper conclusions from his work.

Taken altogether it is a most valuable work.

CHRISTIANITY AND SEX PROBLEMS. By Hugh Northcote, M. A. Crown octavo, 257 pages. Bound in extra cloth. Price, $2.00 net. F. A. Davis Co., Publishers, 1914 Cherry St., Philadelphia.

In a single sentence of their book, two modern biologists have given pregnant expression to one of the most imperative of present day needs. We need, they say, a new ethic of sexes. Such a new ethic is being gradually evolved as the outcome of the thoughts and labors of many, writing with various motives and with a greater or less degree of conscientiousness on the series of problems arising from the physiology and psychology of sex. The book before us is a clean straightforward work and it is recommended to those interested in this subject.

